# Bacteria of the order *Burkholderiales* are original environmental hosts of type II trimethoprim resistance genes (*dfrB*)

**DOI:** 10.1093/ismejo/wrae243

**Published:** 2024-12-10

**Authors:** David Kneis, Faina Tskhay, Magali de la Cruz Barron, Thomas U Berendonk

**Affiliations:** Dresden University of Technology, Institute of Hydrobiology, 01062 Dresden, Saxony, Germany; Dresden University of Technology, Institute of Hydrobiology, 01062 Dresden, Saxony, Germany; Dresden University of Technology, Institute of Hydrobiology, 01062 Dresden, Saxony, Germany; Dresden University of Technology, Institute of Hydrobiology, 01062 Dresden, Saxony, Germany

**Keywords:** antibiotic resistance genes, environment, original host, trimethoprim

## Abstract

It is consensus that clinically relevant antibiotic resistance genes have their origin in environmental bacteria, including the large pool of primarily benign species. Yet, for the vast majority of acquired antibiotic resistance genes, the original environmental host(s) has not been identified to date. Closing this knowledge gap could improve our understanding of how antimicrobial resistance proliferates in the bacterial domain and shed light on the crucial step of initial resistance gene mobilization in particular. Here, we combine information from publicly available long- and short-read environmental metagenomes as well as whole-genome sequences to identify the original environmental hosts of *dfrB*, a family of genes conferring resistance to trimethoprim. Although this gene family stands in the shadow of the more widespread, structurally different *dfrA*, it has recently gained attention through the discovery of several new members. Based on the genetic context of *dfrB* observed in long-read metagenomes, we predicted bacteria of the order *Burkholderiales* to function as original environmental hosts of the predominant gene variants in both soil and freshwater. The predictions were independently confirmed by whole-genome datasets and statistical correlations between *dfrB* abundance and taxonomic composition of environmental bacterial communities. Our study suggests that *Burkholderiales* in general and the family *Comamonadaceae* in particular represent environmental origins of *dfrB* genes, some of which now contribute to the acquired resistome of facultative pathogens. We propose that our workflow centered on long-read environmental metagenomes allows for the identification of the original hosts of further clinically relevant antibiotic resistance genes.

## Introduction

Trimethoprim (TMP) is a synthetic antibiotic inhibiting bacterial DNA and RNA synthesis via disruption of folic acid production [1]. TMP is typically administered as cotrimoxazole, a combination of TMP and sulfamethoxazole, targeting two independent stages of the folic acid metabolic pathway [2].

Nowadays, specific TMP resistance in human and veterinary pathogens is typically mediated by *dfr* genes coding for drug-insensitive dihydrofolate reductases. The genetic context of *dfr* in clinical isolates suggests that horizontal gene transfer is a major driver of the fast proliferation of TMP resistance [3]. Specifically, acquired *dfr* genes are often found in the cargo of class 1 integrons [4,5] which generally host an instance of the *sul1* gene [6]. It is this common co-presence of *dfr*-borne TMP resistance and *sul1*-mediated sulfonamide resistance which explains the failure of cotrimoxazole-based infection therapy in many cases [7].

According to a recent June 2024 snapshot of the CARD database [8], *dfr* genes are distributed across the families *dfrA* and *dfrB* with more than 10 members each and eight further genes indexed *dfr*C through *dfr*L. The *dfrB* family has recently seen an increase from 10 to 20 members not yet reflected by CARD. Phenotypic TMP resistance conferrend by the novel variants has been demonstrated in MIC assays [9,10]. The *dfrB* family stands out from other *dfr* due to its very short sequence (237 bp, 79 amino acids) and the unusual mode of substrate binding by the dfrB enzymes [11].

As demonstrated previously, genes of the *dfrB* family can largely be divided into two major groups based on their genetic context [12]. The early discovered variants *dfrB1* through *dfrB7* are mostly found in a gene context reflecting mobility. Specifically, these variants are typically associated with integrons as inferred from the presence of integrases, gene cassette recombination sites, and/or other markers like the *qacE$\Delta $1* multi-drug efflux pump in close proximity to *dfrB*. This is in contrast to the later discovered *dfrB* variants with a numeric index beyond 8, some of which are almost omnipresent in freshwater systems of temperate regions (Europe, North America, Asia, New Zealand) with *dfrB10* being the predominant variant seen so far [12]. Although the latter study disclosed aquatic microbiomes as a reservoir of *dfrB*-like TMP resistance genes, the original host organism(s) remained identified. Knowledge about the original environmental hosts, however, is essential for understanding resistance mobilization from native environmental microbiomes to human or veterinary pathogens [13]. Based on such knowledge, the risk of resistance mobilization could eventually be minimized by restricting contamination of the primary habitats of the original hosts with pathogen-enriched media like manure or wastewater.

Here, we exploit information from long-read metagenomes and whole genome assemblies to identify several families of the bacterial order *Burkholderiales* as original hosts of *dfrB*-like genes conferring type II trimethoprim resistance. Further, we demonstrate that the high abundance of *dfrB* variants, most prominently *dfrB10*, is not limited to freshwater communities but also pertains to soil habitats, including permafrost.

## Materials and methods

### Abundance of *dfrB* variants in environmental communities

We previously scanned a rich set of public short-read metagenomic sequences covering both freshwater and wastewater environments for the known *dfrB* genes as well as 16S rRNA genes [12]. For this study, we employed the same blast-based workflow but complemented the data set with soil-borne metagenomes (148 data sets from eight countries across four continents). For the sake of uncomplicated comparison with freshwater-related data, only shotgun metagenomic sequences produced in paired-end mode with Illumina short-read technology were included. The average sequence length after merging of read pairs was 249 bp. A complete list of the processed short-read metagenomes, including accession identifiers, can be found in the supplementary Table S1.

As usual, gene counts are reported relative to 16S rRNA genes to facilitate comparisons across samples. Relative abundance was computed as (c_dfrB_ / 237) ^*^ (c_16S_ / 1550) where "c" represents the respective count data and integers are the corresponding representative gene lengths in bp.

### Identification of original hosts of environmental *dfrB*

#### Focus on non-mobile instances

As pointed out in the introduction, several variants of *dfrB* apparently underwent mobilization in the past. It is primarily those integron-bound cases hosted by pathogens that are returned when global standard nucleotide sequence databases are scanned for *dfrB*. However, in line with the widely accepted theory of the origin of resistance [14], we expect the original environmental host(s) to carry non-mobile instances of *dfrB* in their chromosomal DNA as part of, e.g. metabolic gene clusters. Consequently, we focused on the taxonomic identification of bacteria that carry a copy of *dfrB* but lack markers of mobility in genomic neighbourship. As markers of potential mobility we consider signatures of integrons, insertion sequences [15,16], but also other antibiotic resistance genes (ARG) indicating embedding in resistance gene cassette arrays [12].

#### Gathering information on gene context

To reliably assess potential mobility, the *dfrB* genes need to be captured together with a decent amount of flanking DNA comprising many hundreds to several thousands of base pairs. Ideally, sequences would be long enough to also include a phylogenetic marker (e.g. a 16S RNA gene) or several of such markers [17] allowing for direct inference of the host. Currently, culture-free approaches to obtaining long DNA sequences from environmental samples include long-read sequencing and computational assembly methods, with the latter being capable of exploiting short reads too. Metagenome assembly is, however, computationally very demanding and the likelihood of obtaining accurate long contigs is typically low for native environmental samples where limited sequencing coverage meets extraordinary microbial diversity. By means of a seed-based assembly of short-read metagenomic data, we previously reconstructed flanking sequences of *dfrB* genes up to a length of ~1200 bp [12]. Although this length allowed for identifying markers of gene mobility in direct proximity of the *dfrB*, the likelihood of capturing a sequence suitable for host identification (e.g. 16S rRNA genes) remained negligibly low.

Consequently, for the purpose of host identification, we turned towards the analysis of long-read metagenomic sequences available in the sequence read archive (SRA, https://www.ncbi.nlm.nih.gov/sra). Specifically, we analysed sequences identified as metagenomes of original environmental community DNA sequenced using long-read technology (MinIon, GridIon, PromethIon). The corresponding accession numbers and metadata can be found in supplementary Table S2.

### Identification of candidate hosts

All long-read data sets were initially trimmed at head and tail by 50 bp and they were subsequently scanned for the occurrence of the currently known gene variants *dfrB1* to *dfrB21*. For reads with multiple significant alignments, the corresponding *dfrB* variant was taken to be the one indicated by the lowest e-value. The total number of *dfrB*-positive reads was 103 with the vast majority of e-values (95 out of 103) being less than 1e-30. Subsequently, from each of those reads, the subsequence identified as *dfrB* (typically 237 bp) was deleted by marking the corresponding nucleotide identifiers as unknown (nucleotide code „N“). The remaining sequences are subsequently called "flanking sequences" or just "flanks".

The flanking sequences were then aligned against the NCBI nucleotide collection database (nr/nt, version February 2024) by means of "blastn" using a word size of 16, requesting a minimum sequence identity of 75%, a minimum coverage of 10%, as well as an overall cutoff e-value of 1e-30. For each flank, the table of significant alignments was processed through R to clear the genus names linked with all subject sequences from duplicates to obtain a list of unique genera. Genera reported for flanking sequences originating from at least three physically independent samples were subsequently considered as **candidate original hosts**.

### Verification of candidate original hosts

As a primary means of validation, all genome assemblies available in Genbank for the respective candidate original host genus were scanned for both the occurrence of *dfrB* genes and any genetic elements found in proximity of a *dfrB* in the environmental long-read dataset (proximity was defined as the flanking region truncated to a maximum length of 2000 bp). Genomes yielding a significant hit for both a *dfrB* and a second target were considered as verified original hosts unless the second target represented a marker of potential mobility (which was never the case). The matching of GC contents between *dfrB* flanking sequences and the respective assembled genome was also verified. The entire workflow used to identify original *dfrB* hosts from environmental long-read metagenomes is outlined in the supplementary Fig. S1.

As a fully independent means of verification, short-read metagenomic datasets were tested for empirical correlations between the abundance of *dfrB* and the number of 16S rRNA gene copies attributable to the bacterial family comprising most of the verified original hosts.

## Results

### *dfrB-like* genes are common in freshwater and soil microbiomes

**Figure 1:**
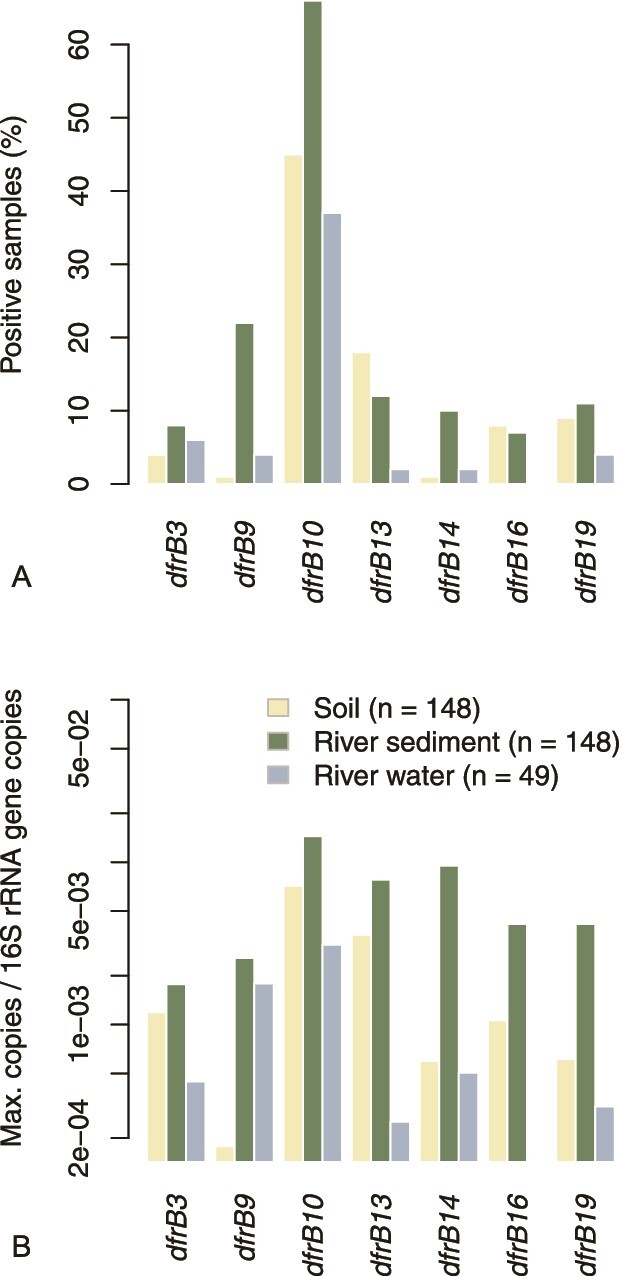
Prevalence of *dfrB* genes in short-read metagenomic samples from different environmental compartments. (a) Percentage of samples tested positive. (b) Highest relative abundance observed. Gene variants detected in less than 5 samples overall have been omitted for clarity in both sub-figures.

Based on short-read metagenomes, we previously identified freshwater bacterial communities as a rich source of *dfrB* genes with a clear predominance of the variant *dfrB10*. The additional evaluation of soil-borne short-read data demonstrates that *dfrB* genes are regularly hosted by terrestrial microbiomes too. Again, *dfrB10* was found to be the variant with the greatest prevalence, indicated by 40% of positively tested samples (Fig. 1A).

Yet the majority of samples was tested negative for *dfrB*-like genes, reflecting their absence in some microbiomes and/or the limited sequencing coverage. Because of this zero inflation, averaged relative abundance data are hardly representative, hence we report observed maximum values instead (Fig. 1B). In general, relative abundances in terrestrial soil communities appear to lag behind those observed in river sediments but differences are marginal in case of the predominant variant *dfrB10* as well as *dfrB3 and dfrB13*.

**Figure 2:**
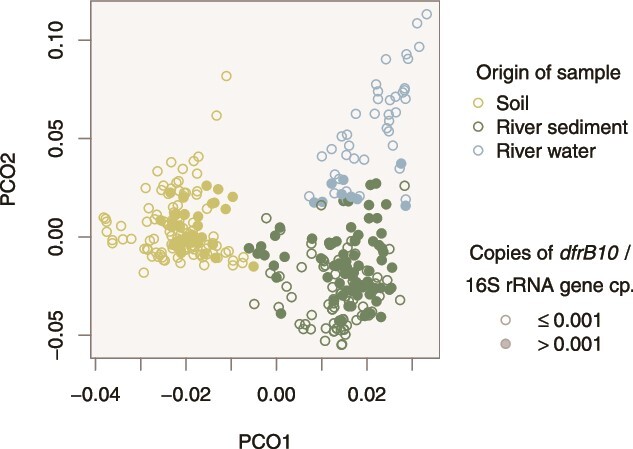
Principal coordinate analysis illustrating the relative abundance of *dfrB10* against the background of bacterial community composition. The latter was evaluated at the level of bacterial families as inferred from 16S rRNA gene classification. Bold symbols highlight samples where the estimated relative abundance exceeds 1:1000 *dfrB10* copies per 16S rRNA gene copies.

Fluvial deposits partly consist of eroded terrestrial soil, giving rise to the hypothesis that the occurrence of *dfrB* in river sediments solely reflects bacterial migration from soil to water triggered by, e.g., surface runoff. A combined analysis of community composition and *dfrB10* gene counts, however, disclosed high relative abundances even in those river sediments which are clearly distinct from terrestrial soils in terms of community composition (Fig. 2). Consequently, hosts of *dfrB* likely reside in both typical soil habitats and aquatic environments, including transition zones. At the same time, a high relative abundance of *dfrB10* in water samples was generally associated with a community composition similar to that of river sediments. Hence, the occurrence of *dfrB10* in river water likely reflects resuspension of bed material rather than the presence of that gene in genuine planktonic bacteria.

### A consistent genetic context of environmental *dfrB* genes

The search for the original host(s) in environmental microbiomes is driven by the fundamental hypothesis that *dfrB* genes are regularly carried by certain phylotypes. Within those phylotypes, we expect the *dfrB* genes to be part of a gene cluster with a specific but possibly unknown function. Consequently, we expect the flanking sequence of any particular instance of a *dfrB* gene to be highly similar to other flanking sequences present in environmental data sets.

To test for the hypothesized similarities, we trimmed the flanking sequences extracted from the long-read metagenomic dataset to cover a radius of at most 2000 bp around the *dfrB*. These "close flanks" were then aligned against each other using a strict similarity filter (cutoff e-value of 1e-30). Indeed, 80% of the close flanking sequences of *dfrB10* genes exhibited significant similarity with other close flanks, indicating the existence of a “typical gene context” (Table 1). For *dfrB* genes other than variant 10, the percentage of close flanking sequences with highly similar homologs was markedly lower but still amounted to ~60%.

**Table 1:** Similarity between close flanks of *dfrB* genes extracted from environmental long-read metagenomes.

*dfrB* gene variant	*dfrB10*	All other *dfrB*
Total close flanks analyzed	78	25
Cases identified as similar to flanks from at least one **independent** metagenomic sample	60 (77%)	12 (48%)
Cases identified as similar to other flanks from the **same** metagenome	2 (3%)	3 (12%)
Cases without similarity	16 (20%)	10 (40%)

Close flanks were defined by a maximum radius of 2000 bp around the *dfrB*. Two close flanks were considered similar if the e-value of a blast-based alignment was at most 1e-30.

### All but one candidate original hosts belong to *Burkholderiales*

**Figure 3:**
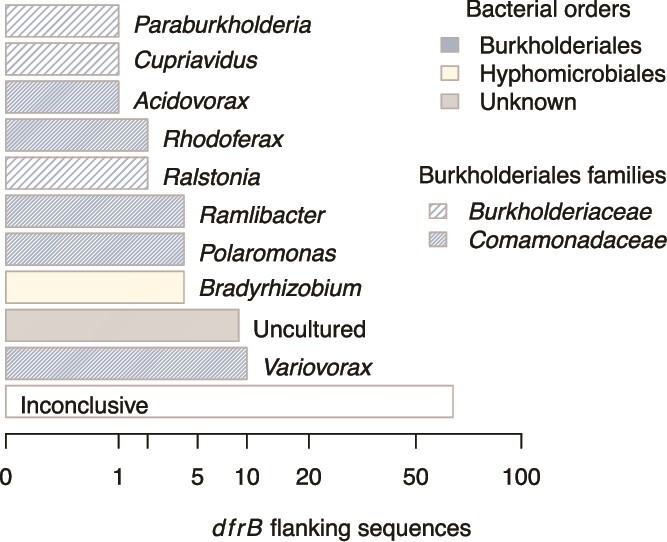
Candidate host genera identified by aligning the 103 flanking sequences of *dfrB* genes against the NCBI standard nucleotide database. Indicated is the number of *dfrB* flanks where the best-matching subject sequence was attributable to the reported genus. For example, the value for *Variovorax* implies that subject sequences attributed to that genus produced the best alignment for 10 of the 103 *dfrB* flanks extracted from long-read metagenomes. Inconclusive cases are disentangled in the supplementary Fig. S3. The association of genomic data of the displayed genera with particular long-read metagenomes (source of the flanking sequences) is documented in Table S4.

The screening of 103 *dfrB* flanking sequences against NCBI's standard nucleotides database resulted in 38 cases where significant alignments met the set quality criteria and the taxonomic origin of the subject sequence was reported for at least three independent samples. Thereof, 29 cases pointed to a particular source genus, whereas, in the other nine cases, homologs were only found in incomplete metagenome assemblies lacking taxonomic information (Fig. 3).

In the remaining 65 cases, no genus information could be assigned to a flanking sequence, either because no significant alignment was found at all, alignments did not meet the adopted quality criteria, or because replication was considered insufficient with the source genus of the subject sequence being reported for less than three samples.

With the exception of *Bradyrhizobium*, all of the candidate host genera reported in Fig. 3 represent members of the families *Burkholderiaceae* or *Comamonadaceae* both belonging to the order *Burkholderiales*. The genus that clearly dominated the best-hit subject sequences was *Variovorax* which comprises motile, typically heterotrophic bacteria with aerobic or facultatively anaerobic metabolism found in a variety of terrestrial and aquatic habitats [18].

### Genome assemblies confirm *Burkholderiales* as an original environmental host order

To validate the set of candidate original hosts as shown in Fig. 3, we analyzed all genome assemblies for the respective genera available in Genbank as of May 2024. These genomes were scanned for the presence of *dfrB* genes and similarities with the close *dfrB* flanking sequences extracted from the long-read metagenomes. Simultaneous hits for both *dfrB* and flank markers were obtained for genomes representing *Variovorax*, *Polaromonas*, *Ramlibacter*, and *Acidovorax*. All four genera belong to the *Comamonadaceae* family (Table 2).

**Table 2:** Assembled genomes of candidate original hosts harboring a *dfrB* gene with indication of the genetic context.

			Detected *dfrB*			Similarity with *dfrB* flank	
Accession (GenBank)	Genus / species	Gene	Identity (Nucl.)	Identity (AA)	Cover-age	e-value	Found where
GCA_031422495.1	*Polaromonas* sp.	*dfrB10*	94.5	94.9	100	5E-10	Any contig
GCA_029945485.1	*Polaromonas* sp.	*dfrB10*	95.3	97.4	98	2E-21	Contig with dfrB
GCA_002413825.1	*Rhodoferax* sp.	*dfrB10*	97.0	100.0	100	1E-92	Contig with dfrB
GCA_018780565.1	*Rhodoferax* sp.	*dfrB10*	94.9	97.4	100	2E-13	Contig with dfrB
GCA_031365665.1	*Rhodoferax* sp.	*dfrB10*	94.5	98.3	76	4E-41	Contig with dfrB
GCA_030317115.1	*Variovorax* sp.	*dfrB10*	91.1	89.7	99	6E-22	contig with dfrB
GCA_030317065.1	*Variovorax* sp.	*dfrB10*	95.8	98.7	100	< 1E-100	Any contig
GCA_030316825.1	*Variovorax* sp.	*dfrB10*	95.8	98.7	100	< 1E-100	Contig with dfrB
GCA_031454625.1	*Variovorax* sp.	*dfrB10*	95.4	97.4	100	2E-12	contig with dfrB
GCA_031454625.1	*Variovorax* sp.	*dfrB10*	92.3	80.0	99	< 1E-100	Any contig
GCA_003797765.1	*Ramlibacter* sp.	*dfrB13*	96.2	96.2	100	< 1E-100	contig with dfrB
GCA_028292245.1	*Ramlibacter* sp.	*dfrB19*	87.2	83.3	99	< 1E-100	Contig with dfrB
GCA_028292185.1	*Ramlibacter* sp.	*dfrB19*	88.2	83.3	100	4E-48	Any contig
GCA_035367235.1	*Rhodoferax* sp.	*dfrB19*	88.6	84.6	100	–	–
GCA_016718155.1	*Rhodoferax* sp.	*dfrB19*	91.2	84.6	100	2E-74	Contig with dfrB
GCA_035910795.1	*Rhodoferax* sp.	*dfrB19*	89.0	84.6	100	3E-43	Any contig
GCA_035913435.1	*Rhodoferax* sp.	*dfrB19*	88.7	84.6	100	8E-56	Any contig

Identity and query coverage are given in %. Dashes indicate a lack of significant alignment between the assembled genome and any of the close *dfrB* flanking sequences extracted from long-read metagenomes. None of the assembles genomes contained any other specific antibiotic resistance gene.

**Figure 4:**
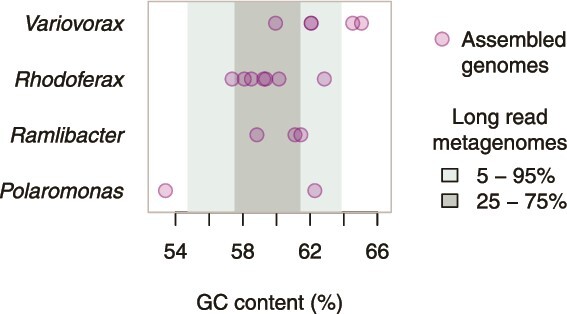
GC content of the DNA in proximity to *dfrB* genes in assembled genomes (Table 2) and long-read environmental genomes. The long-read data are represented by colored ranges covering 50% and 90% of the observed *dfrB* gene instances reported in the supplementary Table S2. Proximity was defined as a radius of 2000 bp.

To further consolidate evidence provided by Table 2, we compared the local context of *dfrB* in the genome assemblies and the long-read metagenomes with regard to GC content (Fig. 4). With an average of 60% (57–65) for the genomes and 59% (55–64) for the long read sequences, the GC content in proximity to *dfrB*-like genes was very similar (*P* > 0.2, values in brackets are 5%–95% quantiles).

Although Table 2 clearly identified bacteria of the order *Burkholderiales* as original environmental hosts of *dfrB* genes, we cannot exclude the existence of further original hosts in other branches of the phylogenetic tree. To possibly identify such other branches, we scanned a larger collection of additional genome assemblies. To keep the computational load within bounds and allow for a careful by-eye inspection of results, we followed a staged approach: [1] First, we screened all NCBI reference bacterial genomes (n = 19 695) for *dfrB*-like genes to pragmatically cover the entire bacterial domain. In this data set, each known species is typically represented by a single whole genome assembly. [2] Second, we performed a deeper screening in the class *beta-Proteobacteria* by scanning all NCBI-annotated assemblies regardless of the level of completeness (n = 17 399). The assemblies in this data set comprise whole genomes and plasmids but also partly assembled material (contigs and scaffolds). This particular screening was done to exhaustively cover all branches of the phylogenetic having the same root branch (i.e. class) as the order *Burkholderiales*. [3] Finally, we performed a screening of assemblies originating from *alpha*- and *gamma-Proteobacteria*. The rationale was to also cover the other major branches under the phylum *Proteobacteria* in greater detail. Since the class *gamma-Proteobacteria* comprises so many human and veterinary pathogens, it is represented in the genome databases with a very rich set of assemblies. Consequently, for the sake of feasibility, we restricted the analysis to NCBI-annotated assemblies flagged as complete, i.e. whole genomes and entirely sequenced plasmids (n = 21 391).

Out of the 58 485 scanned assemblies, a total of 155 contained sub-sequences exhibiting significant, high quality alignments to the currently known *dfrB* genes (quality criteria: e-value ≤ 1e-30, ≥ 90% coverage of subject gene, ≥85% sequence identity at nucleotide level). These 155 assemblies were subsequently inspected for the genetic context of the *dfrB* gene with a focus on indicators of mobility.

In about 75% of the cases (118 of 155 assemblies), the *dfrB* gene was embedded in a class 1 integron (Table S3). Since class 1 integrons are known to be of the major structures involved in ARG capturing and spreading, including plasmid-mediated lateral transmission, these cases do *not* provide useful information for the identification of original hosts. These assemblies rather reflect the spectrum of hosts already having acquired type-II trimethoprim resistance.

The remaining 25% of the cases (37/155), where *dfrB* was *not* evidently part of a class 1 integron, were exclusively attributed to assemblies of genomic material from bacteria classified as *Burkholderiales* (Table S3). Hence, neither the screening of genome assemblies across the whole bacterial domain nor the intensified scanning within the phylum of *Proteobacteria* suggested any additional candidate original hosts of *dfrB*, besides *Burkholderiales*.

### Statistical support from short-read metagenomic datasets

**Figure 5:**
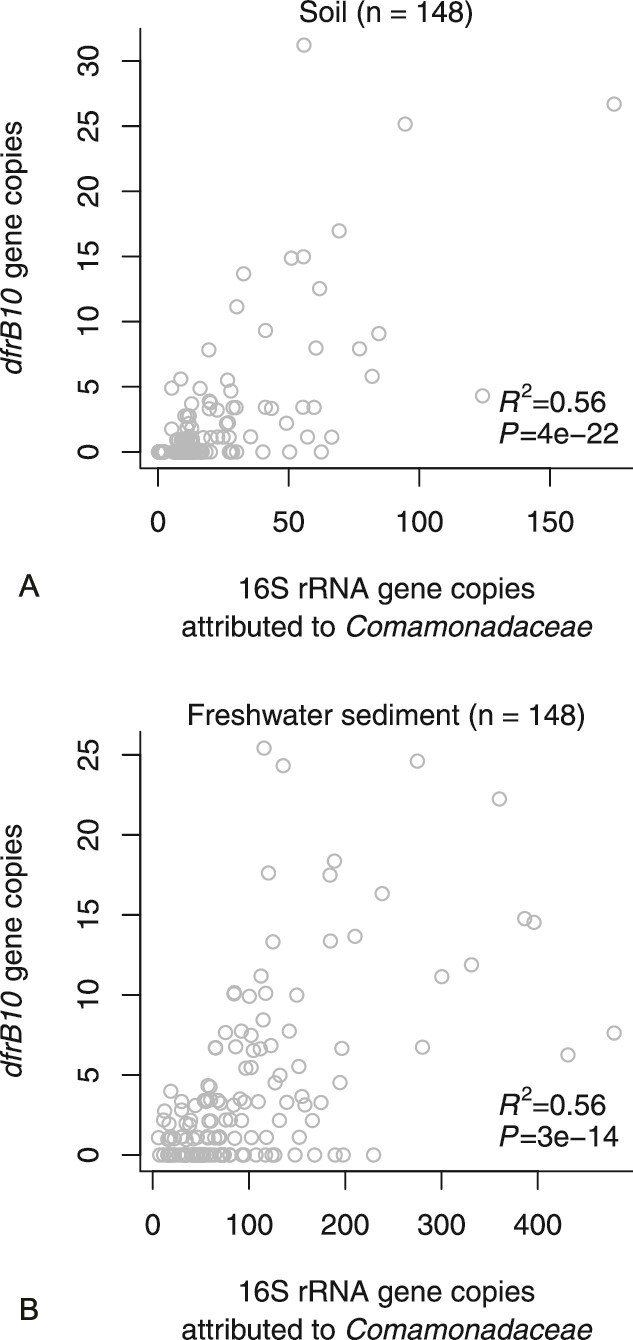
Gene copies of *dfrB10* in relation to the corresponding number of 16S rRNA gene copies attributed to the bacterial family *Comamonadaceae* (order *Burkholderiales*). The analysis is based on short-read metagenomic sequences of soil (a) and freshwater (b) from a multi-continent dataset (Table S1). Simultaneous equality of *R*^2^ and sample size is by coincidence; *P* values refer to a Pearson correlation test.

If bacteria of the order *Burkholderiales* in general and *Comamonadaceae* in particular actually serve as major original hosts, we expect environmental metagenomic DNA samples to exhibit a positive correlation between the abundances of *dfrB* and taxonomic markers like family-related 16S rRNA genes. The short-read environmental metagenomes support this expectation since moderate but highly significant correlations were found in soil-borne and river sediment samples (Fig. 5) as confirmed by partial *R*^2^ analysis. In soil, the average relative abundance was 0.12 *dfrB10* copies per 16S rRNA gene copy attributable to *Comamonadaceae*. In river sediments, the value was about three times lower (0.04). Considering that the median copy number of 16 s rRNA genes in *Comamonadaceae* is 3 (range 1–11) [19], the data suggest that the *dfrB10* gene is carried by a minor fraction of bacteria within that family.

## Discussion

### *Burkholderiales* as confirmed original environmental hosts

Our analysis of both short- and long-read metagenomes clearly demonstrates the omnipresence of *dfrB* genes in both aquatic and terrestrial environmental compartments with relative abundances reaching values as high as 10^−3^ copies per 16S rRNA gene copies. The evaluation of long-read sequences clearly confirms recent findings [12] according to which environmental instances of *dfrB* lack the common genetic context associated with acquired ARGs (e.g. transposons, or integrons) reflecting their mobility. At the same time, the genetic context of *dfrB* was found to be largely consistent across samples, indicating an embedding in conserved regions of bacterial genomes. Consequently, the reads of environmental metagenomes which harbor a copy of *dfrB* most likely represent DNA fragments of the original environmental host(s).

By analyzing the *dfrB* flanking regions of those reads, we identified the order *Burkholderiales* in general and the family *Comamonadaceace* in particular as branches of the phylogenetic tree which comprise several candidate original hosts (Fig. 3). We successfully confirmed four of the candidate genera based on whole genome sequences (Table 2). Moreover, the presence of *Comamonadaceace* was shown to be a good predictor of *dfrB* abundances in independent metagenomic data sets (Fig. 5). Hence, this bacterial family and order deserve qualification as confirmed environmental hosts.

Given that original hosts have been identified for <5% of the acquired ARGs so far [13], our finding contributes a small but important piece to a large puzzle. Nevertheless, even for the specific case of *dfrB*, several questions remain to be answered.

### A single original host order?

Based on current knowledge, it remains difficult to judge whether *Burkholderiales* are the one and only original reservoir of *dfrB* or whether further bacterial orders harbor a yet unidentified set of additional original hosts. Since reliable whole genome sequences are lacking for the vast majority of environmental bacteria as a result of non-culturability, the question may not find an exhaustive answer anytime soon. Nevertheless, our results allow for a pragmatic assessment.

A significant proportion of the *dfrB* flanking sequences did not exhibit significant similarity to any subject in NCBI's nucleotide reference database making it impossible to predict a taxonomic origin. This may be seen as an indication of additional, yet hidden original hosts which are not covered in the database either because of non-culturability or because the respective species have never been in the focus of isolation and whole-genome sequencing attempts. However, all but one of the *dfrB* flanking sequences present in the genomes of *Comamonadaceae* isolates (Table 2) exhibit significant similarities with the flanking sequences extracted from environmental long-read metagenomes. In other words, the *dfrB* context found in those *Comamonadaceae* isolates is not exceptional but apparently representative for the studied source environments. Hence, *Burkholderiales* in general, and *Comamonadaceae* in particular represent a main environmental reservoir of *dfrB* rather than being the tip of an iceberg of unidentified hosts. This view was corroborated by the screening of the about 20.000 NCBI reference genomes and close to 40.000 *Proteobacteria*-borne assemblies for *dfrB* genes followed by discrimination between acquired and apparently immobile cases (Table S3). The screening revealed no evidence for original hosts of *dfrB* in branches of the phylogenetic tree other than the *Burkholderiales* branch.

### Mobilization from the original hosts

Given that *Burkholderiales* are (at least part of) the main original reservoir of *dfrB*, this group likely stands at the origin of proliferation pathways reaching up to contemporary pathogens which often carry a *dfrB* gene alongside other ARGs. Tracing these pathways is, however, challenging. According to common understanding, there must have been initial mobilization event(s) mediated by small transposable elements like insertion sequences [20]. Those elements would be key to the integration of *dfrB* in vectors allowing for a subsequent across-genome proliferation, namely plasmids, where variants like *dfrB10* were originally discovered [21].

Some of the close flanking regions of *dfrB* recovered from the long-read metagenomes indeed contain signatures of insertion sequences (IS), belonging to the families IS3 and IS1595 (two instances each) or IS256 (single instance). In 4/5 of the cases, the best sequence alignments were obtained for IS first detected in the *Comamonadaceae* family (ISVasp3, ISCte2m, and ISAav4) which is in line with the evidence provided above regarding host associations. Whether these IS actually played a role in *dfrB* mobilization remains to be explored.

A closely connected question concerns the diversity of the known *dfrB* variants (Fig. S2). Did diversification occur in the original environmental hosts (e.g. *Burkholderiales*) already, or did the variants diverge only after their mobilization and transmission to new hosts? On the one hand, several of the 20 known gene variants are highly abundant in environmental metagenomes (Fig. 1) but they are rarely reported in clinical isolates. By contrast, variants like *dfrB1*, *dfrB2*, *dfrB4*, and *dfrB5* are frequently found in clinical isolates of facultative pathogens [9] (Table 2) and human-associated wastewater [12] but they are hardly detectable in soil and freshwater metagenomes. Thus, the present data suggest that diversification may have happened simultaneously in the original environmental hosts and in the carriers of acquired *dfrB*-like genes.

### What is the original function of DfrB?

Unraveling the presence and proliferation of *dfrB* in contrasting microbiomes requires comprehension of the corresponding protein function. In spite of in-depth structural information, however, the selective advantage of DfrB in relation to the common dihydrofolate reductase FolA found in most bacteria remains poorly understood. Even though specific bacterial strains are known to rely on alternatives to FolA [22], this does not apply to the identified host genera like, e.g. *Variovorax*, in general. At the same time, previous studies concluded that resistance to the synthetic drug trimethoprim is unlikely to serve as the original selection factor in environmental bacteria [10]. Although the resistance provided by *dfrB* certainly provides an advantage under drug exposure, the true story of proliferation may go beyond simple deterministic principles. For example, a class 1 integron harbored by a *Pseudomonas putida* isolate (NCBI RefSeq assembly GCF_036347735.1) contains a copy of *dfrB1* next to the non-homologous *dfrA1*, both of which code for TMP resistant dihydrofolate reductases. On the one hand, cases like this may simply reflect stochasticity in the process of ARG acquisition. On the other hand, they suggest that *dfrA* and *dfrB* may actually complement each other if, for instance, the two enzymes exhibit different cost–benefit ratios in distinct environmental settings.

**Figure 6:**
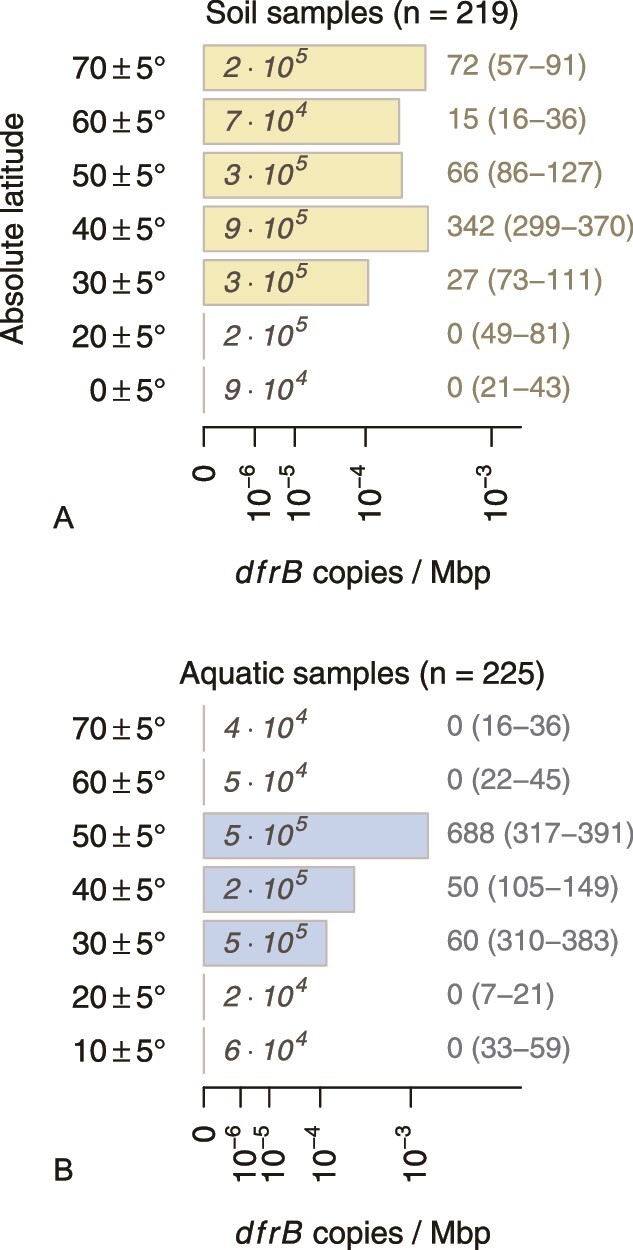
*dfrB* genes in environmental metagenomic sequences (copies per Megabasepairs) in relation to absolute latitude of the sampling location. Short- and long-read sequences (Tables S1 and S2) have been merged for this analysis; aquatic samples comprise both water and sediment. Italic numbers represent the amount of sequenced material in the respective latitude bin (Mbp). Integers on the right denote the number of observed gene copies (first number) followed by expected numbers under the null hypothesis of a globally uniform distribution (95% confidence interval of a binomial model).

In recent work, DfrB enzymes were characterized as particularly slow in comparison to FolA with regard to hydride transfer rate constants [11]. Consequently, *dfrB* should prevail in conditions where rapid nucleotide synthesis, a basis of fast growth, is not relevant. This interpretation would be consistent with the fact that some of the confirmed original hosts genera are frequently reported from cold habitats including aquifers, alpine, and polar regions. Whereas *Polaromonas* is described as psychrophilic [23], *Rhodoferax* and *Variovorax* are psychrotolerant at least [18,24]. These phenotypic features match well with the fact that long-read metagenomes of Greenland permafrost soil were found to be particularly rich in *dfrB10* (SRA accession numbers ERR5029615, ERR5029616). In general, the relative abundance of *dfrB* genes in environmental metagenomes was found to be elevated in samples from mid to high latitudes representing temperate to cold climates. By contrast, *dfrB* genes have not been detected in aquatic or soil metagenomes of tropical origin (< 25° latitude) and this trend is very unlikely to merely result from unequal sampling effort (Fig. 6). Consequently, temperature as a potential selective factor should deserve attention in future research addressing the evolution and spread of *dfrB*-like genes.

### Implications

The identification of the original hosts of ARG is a first but important step towards the disclosure of mobilization events and proliferation routes. In particular, knowledge of the original hosts allows bacterial communities to be identified which likely serve as primary ARG donors when brought in contact with potential recipient pathogens through, e.g. wastewater disposal or organic fertilization. Finally, with the example of *dfrB*, our study highlights the need to minimize antibiotic pollution in all compartments, terrestrial and aquatic, to mitigate selection for antibiotic resistance not only in pathogens but also in the original environmental reservoirs.

## Supplementary Material

supplement_fs_wrae243

Table_S1_fs_wrae243

Table_S2_fs_wrae243

Table_S3_fs_wrae243

Table_S4_fs_wrae243

## Data Availability

All analyzed sequence data are publicly available in the sequence read archive (https://www.ncbi.nlm.nih.gov/sra). See the supplementary Tables S1 and S2 for accession numbers.
