# Supplementary material for: Bacteria of the order Burkholderiales are original environmental hosts of type II trimethoprim resistance genes (dfrB)
Source: ISME J. 2024 Dec 10;18(1):wrae243. doi: 10.1093/ismejo/wrae243 (PMC11662351; doi:10.1093/ismejo/wrae243)
Supplement: supplement_fs_wrae243 [file supplement_fs_wrae243.pdf]

Supplementary material

Corresponding article in the ISME journal: „Bacteria of the order Burkholderiales are original environmental hosts of type II trimethoprim resistance genes (*dfrB*)“

by David Kneis [1], Faina Tskhay [1], Magali de la Cruz Barron [1], Thomas U. Berendonk [1]
[1] Dresden University of Technology, Institute of Hydrobiology, 01062 Dresden, Saxony, Germany
david.kneis@tu-dresden.de

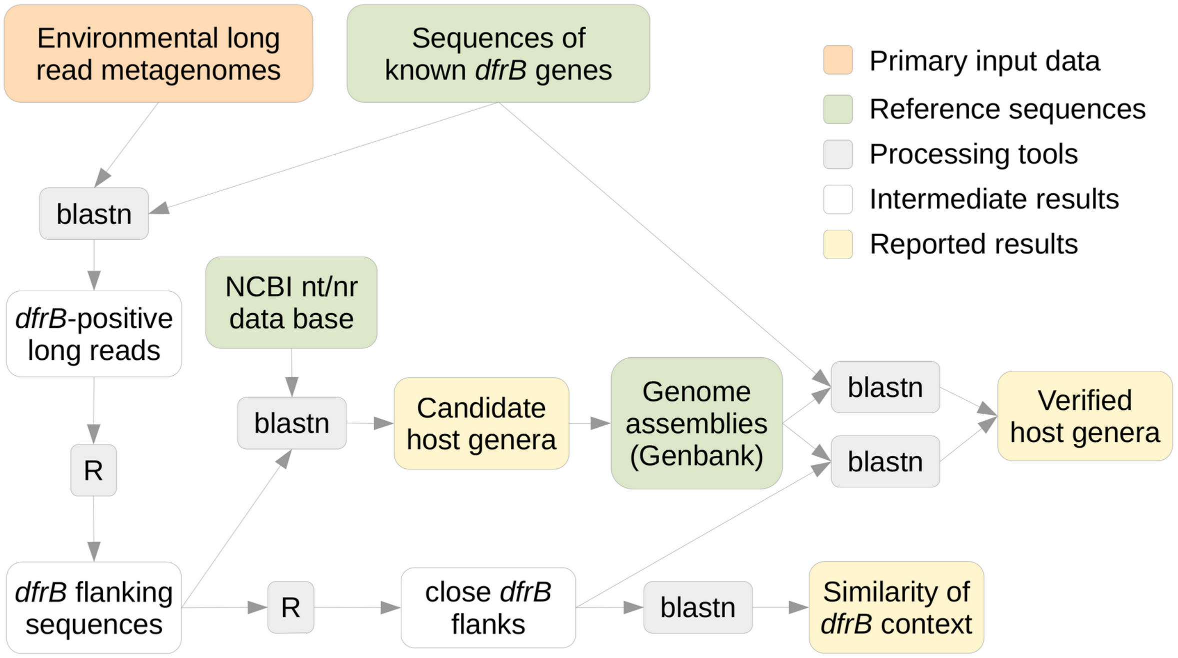

Figure S1: Workflow for the identification of original environmental hosts from environmental long-read metagenomes. "R" indicates script-based processing using the R language and environment for statistical computing (https://www.r-project.org/).

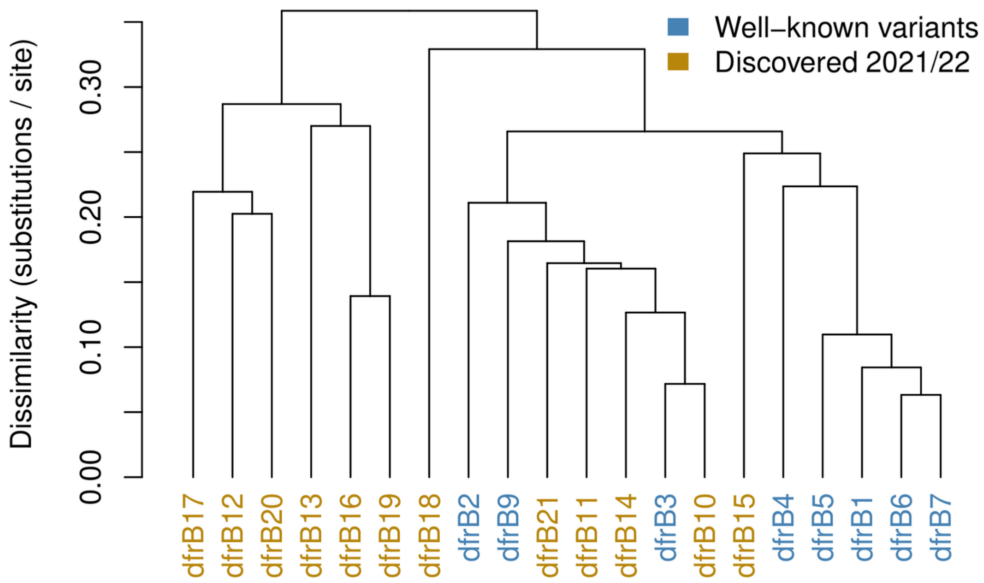

Figure S2: Dissimilarity of the *dfrB* genes based on mismatches in nucleotide sequences. A value of 0.1 represents 1 mismatch per 10 bases.

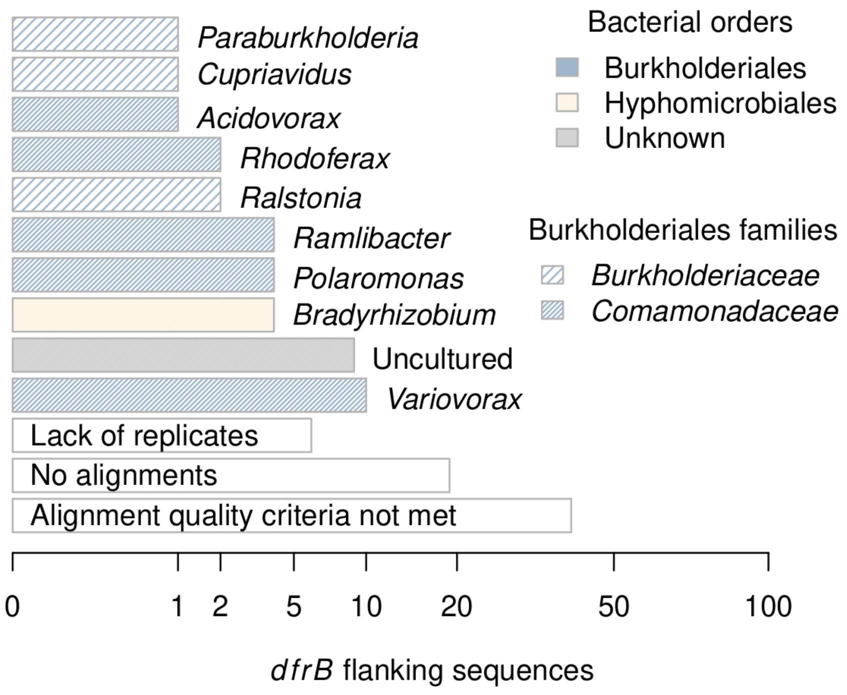

Figure S3: Like Fig. 3 of the main text but with decomposition of the inconclusive cases (unfilled bars at bottom).

Table S1: Listing of the analyzed short-read metagenomic DNA sequences of environmental origin with accession numbers and available metadata. All datasets are available for free download in the sequence read archive (https://www.ncbi.nlm.nih.gov/sra).

The table is provided as a separate tab-delimited plain text file (Table_S1.txt).

Table S2: Listing of the analyzed long-read metagenomic DNA sequences of environmental origin with accession numbers and available metadata. All datasets are available for free download in the sequence read archive (https://www.ncbi.nlm.nih.gov/sra).

The table is provided as a separate tab-delimited plain text file (Table_S2.txt).

Table S3: Genome assemblies accessible through https://www.ncbi.nlm.nih.gov/datasets/genome exhibiting high-quality alignments with known *dfrB* gene variants. For each hit, the gene variant is reported along with the immediate genetic context, host information, and indicators of alignment quality both at nucleotide and aminoacid level.

The table is provided as a separate tab-delimited plain text file (Table_S3.txt).

Table S4: Association of genomic data sets behind the genera displayed in Fig. 3 with particular long-read metagenomes from which *dfrB* flanking sequences were recovered.

The table is provided as a separate tab-delimited plain text file (Table_S4.txt).
